# The effect and stability of MVCT images on adaptive TomoTherapy

**DOI:** 10.1120/jacmp.v11i4.3229

**Published:** 2010-07-02

**Authors:** Poonam Yadav, Ranjini Tolakanahalli, Yi Rong, Bhudatt R Paliwal

**Affiliations:** ^1^ Department of Human Oncology University of Wisconsin ‐ Madison Madison WI; ^2^ Department of Medical Physics University of Wisconsin ‐ Madison Madison WI; ^3^ University of Wisconsin Riverview Cancer Centre Wisconsin Rapids WI; ^4^ Vellore Institute of Technology University Vellore Tamil Nadu India

**Keywords:** TomoTherapy, IVDT, target degradation, MVCT

## Abstract

Use of helical TomoTherapy‐based MVCT imaging for adaptive planning is becoming increasingly popular. Treatment planning and dose calculations based on MVCT require an image value to electron density calibration to remain stable over the course of treatment time. In this work, we have studied the dosimetric impact on TomoTherapy treatment plans due to variation in image value to density table (IVDT) curve as a function of target degradation. We also have investigated the reproducibility and stability of the TomoTherapy MVCT image quality over time. Multiple scans of the TomoTherapy “Cheese” phantom were performed over a period of five months. Over this period, a difference of 4.7% in the HU values was observed in high‐density regions while there was no significant variation in the image values for the low densities of the IVDT curve. Changes in the IVDT curves before and after target replacement were measured. Two clinical treatment sites, pelvis and prostate, were selected to study the dosimetric impact of this variation. Dose was recalculated on the MVCTs with the planned fluence using IVDT curves acquired before and after target change. For the cases studied, target replacement resulted in an overall difference of less than 5%, which can be significant for hypo‐fractionated cases. Hence, it is recommended to measure the IVDT curves on a monthly basis and after any major repairs/replacements.

PACS numbers: 87.55.Qr, 87.56.bd, 87.57.C, 87.57.Q

## I. INTRODUCTION

It is more than two decades since 3D conformal radiotherapy that a forward planning methodology was introduced in all modern radiotherapy departments. In 1982, Anders Brahme(1) for the first time proved analytically a possibility of inverse planning to maximize dose to a target while minimizing dose to the neighboring critical structures. Based on this concept, most of the radiotherapy departments practice intensity‐modulated radiotherapy (IMRT) for better target coverage and tumor control. Requirements of quality assurance (QA) for IMRT are very stringent. As a result, most radiation therapy machines are equipped with online imaging for image‐guided radiotherapy (IGRT) and adaptive radiotherapy (ART). ART is a radiation treatment process where the subsequent delivery can be modified using a feedback of the geometric and dosimetric information from previously treated fractions. It is a multistep process involving dose reconstruction,^(^ ^2^ ^)^ dose accumulation,^(^ ^3^ ^)^ treatment evaluation, recontouring and reoptimization.^(^ ^4^ ^)^ ART uses the MVCT data acquired at the time of treatment, as well as information obtained during delivery verification. It is important that the information from MVCT be directly proportional to the photon attenuation in order to use the images for treatment planning. It has been shown that MVCT images can be used for dose calculation, the accuracy of which is similar or even superior to that of the initial dose calculation based on kVCT images.^(^ ^5^ ^)^ For accuracy of dose calculation, reproducibility and stability of image value to density data are highly important.

The performance of the IGRT systems has been studied extensively in recent years. Performance characteristics of MVCT on Hi·Art TomoTherapy system^(^ ^6^ ^,^ ^7^ ^)^ have been reported. These reports include image noise and uniformity, spatial resolution, contrast properties, and multiple scan average dose from multiple scans. It has been shown that for equivalent doses, MVCT has equivalent spatial resolution and noise characteristics as that of kVCT images. However, kVCT systems outperform MVCT in terms of low contrast visibility. To date, there have not been many studies that track these parameters over time. Recently, Stock et al.^(^ ^8^ ^)^ have investigated and compared only image quality parameters for multislice CT, linac based cone‐beam CT (CBCT) and simulator over a period of 16 months. The image parameters studied were noise, spatial resolution, low contrast visibility (LCV) and uniformity. They reported no significant trend in any of the three devices in their study. Langen et al.^(^ ^9^ ^)^ tested the stability of the MVCT numbers by determining the variation of this calibration with spatial arrangement of the phantom and MVCT acquisition parameters. They found that the largest difference in any of the dosimetric endpoints was 3.1% but, more typically, the dosimetric endpoints varied by less than 2%. They also investigated the variation in the MVCT numbers over a period of nine months, but have not reported dosimetric effects of the IVDT variation over time.

Helical TomoTherapy MVCT uses slip rings to allow delivery of continuous radiation from the rotating gantry. During MVCT imaging, the nominal energy of the incident electron beam is reduced from 6 to 3.5 MeV and the field width (IEC‐Y) of 4 mm is used for the MVCT beam during image acquisition.^(^ ^10^ ^)^ As a standard, helical TomoTherapy MVCT has been used routinely for daily patient treatments setup.^(^ ^6^ ^–^ ^9^ ^,^ ^11^ ^)^ The incorporation of daily images into the radiotherapy process is essential for ART.^(^ ^12^ ^)^

On the Hi·Art system, due to target degradation, X‐ray target replacement is required approximately every 10–12 months. Over time, the target thins initially causing the beam to be more forward peaked and, towards the end of target life, the target thinning causes a decrease in the beam energy and a softening of the beam profile at the lateral edges of the beam. Staton et al.^(^ ^13^ ^)^ studied the effects of target degradation on IMRT delivery. They have reported dosimetric differences of about 4% towards the end of the target life. For dosimetry purpose, the MVCT numbers, or pixel values, need to be converted to electron densities using a CT to electron density calibration curve.^(^ ^14^ ^)^ While the establishment of a CT to electron density curve is straightforward with an appropriate phantom, the variation of this calibration with time needs to be investigated since it impacts dosimetric uncertainties. The overall purpose of this study is to evaluate the dosimetric effects of variation in image value to density table (IVDT) due to target degradation. We have tabulated the variation of IVDT over a period of five months and studied its dosimetric impact on treatment plans for two clinical sites: prostate and pelvic. We also have included in this study the change in IVDT due to relocation of the machine after the relocation beam was matched to the “gold standard”. A similar process is followed after every target replacement. The dosimetric effect of the above actions on clinical cases was studied. In addition, we also present the reproducibility and stability of the TomoTherapy MVCT imaging system with respect to noise and contrast to noise ratio (CNR).

## II. MATERIALS AND METHODS

### A. Stability of image value to density table

MVCTs potentially provide greater accuracy for radiation therapy dose calculations and inhomogeneity corrections.^(^ ^5^ ^)^ The stability of the CT to electron density calibration is an indicator of the CT number integrity and a prerequisite for dose recalculation.

**Figure 1:**
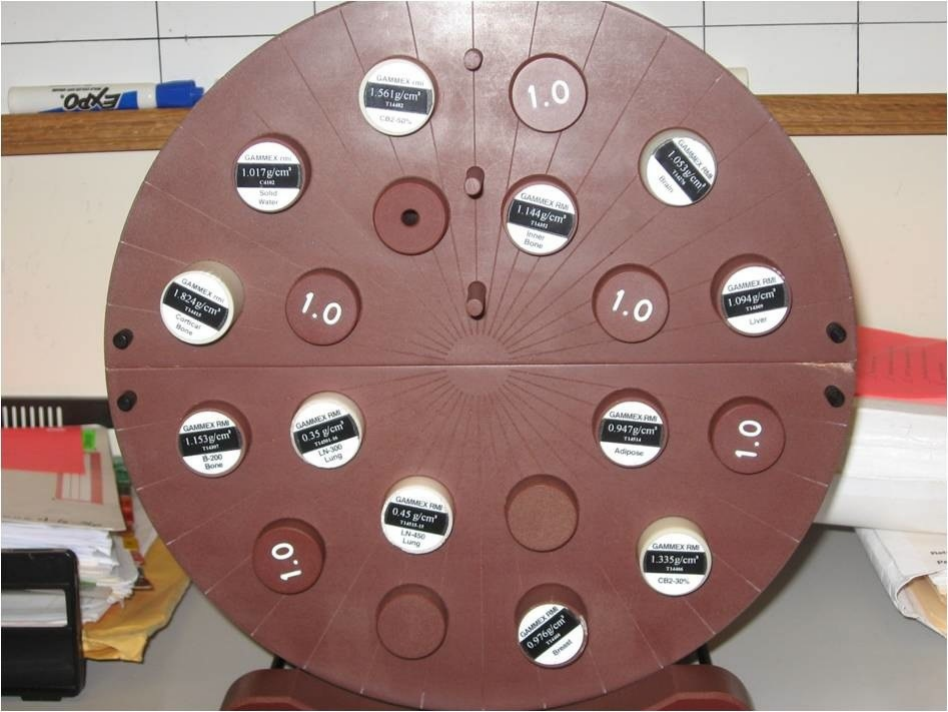
Picture of “Cheese” phantom with plugs of known electron densities inserted into the built‐in twenty cavities.

To establish CT to electron density calibrations, TomoTherapy “Cheese” phantom (Gammex RMI, Middelton, WI) was used. The phantom is an 18 cm thick solid water cylinder with a diameter of 30 cm. The phantom consists of two semi‐cylindrical halves, in between which a film can be placed. There are 20 plugs, including four solid water plugs plus 16 tissue substitute plugs that range in electron density relative to water from $0.29g/{\text{cm}}^{3}$ to $4.59g/{\text{cm}}^{3}$. These can be inserted into 28 mm diameter holes in the “Cheese” phantom. The inserted plugs as shown in Fig. 1 are representative of the range of inhomogeneities observed in the clinical environment. The “Cheese” phantom was scanned multiple times over a period of five months which included an X‐ray target change. Hence, the data provided compare the performance of MVCT towards the end of the target life to that taken after a target replacement. The MVCT images acquired were then imported to the Pinnacle^3^ 8.1 treatment planning system (TPS) (Philips Medical Systems, Fitchburg, WI) to measure image values. Regions of interest with diameter of 20 mm were contoured at the center of each of the phantom plugs and the mean HU values within the contours were recorded. The electron densities of each phantom plug were recorded from the manufacturer specifications, and the physical density corresponding to the mean CT values recorded was plotted as the IVDT curve.

A potential change in IVDT curve is due to changes in beam characteristics caused by X‐ray target wear. A Hi·ART TomoTherapy system requires X‐ray target replacement approximately every 10–12 months. The IVDT curve was recorded for a period of five months over the life of the target. Near the end of target life, the target thins resulting in beam softening and changes in the beam profile at the lateral edges of the beam of up to 6%, as shown by Langen et al.^(^ ^9^ ^)^ To study the changes in the IVDT curve with respect to the target change, the “Cheese” phantom was scanned before and after the target change.

A system move was also performed during the five months period. The system was recommissioned to match the “gold model” treatment beam. The system experienced a table top/couch upgrade and a corresponding software upgrade, which accounts for the changes in the couch. However, no other major parts in the beam line were replaced. Changes in the IVDT curve before and after the system move were also tabulated.

### B. Dose recalculation

The effect of IVDT curve variability was studied on two clinical cases: prostate and pelvic. The treatment plan parameters (prescription, number of fractions, field width, pitch, modulation factor and sinogram) for each plan are shown in Table 1. Each case had one kVCT image (called reference image) that was used as a planning CT. MVCT images (20–40) were acquired for daily registration purposes. A very low dose (approximately 2 cGy) was delivered for each MVCT dataset. The MVCT was acquired in the normal mode with a slice thickness of 4 mm. A treatment plan with appropriate tumor coverage and minimum dose to sensitive structures was planned on kVCT. The dose distribution based on the daily MVCT images was calculated using Planned Adaptive module on TomoTherapy Planning Station with different IVDT curves. The MVCT of the patient acquired just prior to treatment was used for recalculation. The MVCT images were aligned with the kVCT images using the rigid‐body alignment tool in the TomoTherapy image registration panel.^(^ ^15^ ^)^ Then, the intended fluence pattern was used to recalculate the dose distribution on the MVCT image set using the corresponding IVDT curves. Dose recomputations for each case were recalculated for the acquired IVDT curves: prior to target change (end of target life), post target change, before system move, after system move and recommissioning.

**Table 1:** Treatment‐planning parameters: prescription, number of fraction, field width, pitch and planning modulation factor, and sinogram segments for prostate and pelvis.

*Site*	*Prescription*	*Number of Fractions*	*Field Width (cm)*	*Pitch*	*Planning Modulation Factor*	*Sinogram Segments*
Prostate	70 Gy to 99% of the PTV	28	2.5	0.43	2	1.7
Pelvic	45 Gy to 99% of the PTV	25	2.5	0.29	2	5.6

The largest variation in the MVCT calibration curve was found for the high‐density values and this is demonstrated more in the results section. The existence of this effect prompted us to select the PTV in the pelvis case proximal to the pelvic bones. Changes in the dose volume histograms (DVHs) and the dose distribution due to changes in IVDT were used to compare the recalculated plans. DVH points such as $D_{99}$ (dose to 99% of target volume), $D_{5}$ (dose to 5% of target volume) for the planning target volume (PTV), and $D_{50}$ (dose to 50% of target volume) and $D_{5}$ for the critical structures were calculated for all plans. In addition, target coverage (TC) and Homogeneity Index (HI) for the PTVs were calculated as explained below.

Target Coverage: TC describes the fraction of the target volume receiving at least the prescription dose and is defined as:

(1) $$TC=\frac{V_{T,presc}}{V_{T}}$$

For perfect coverage, TC equals 1.0.

Homogeneity Index: Dose homogeneity in the target volumes was quantified by the HI, as recommended by the International Commission on Radiation Units and Measurements. The HI is defined as the greatest dose delivered to 2% of the target volume $\left(D_{2}\right)$ minus the dose delivered to 98% of the target volume $\left(D_{98}\right)$ divided by the median dose $\left(D_{\text{median}}\right)$ of the target volume:^(^ ^16^ ^)^

(2) $$HI=\frac{D_{2\%}‐D_{98\%}}{D_{median}}$$

Smaller values of HI correspond to more homogenous irradiation of the target volume. A value of zero corresponds to absolute homogeneity of dose within the target.

### C. MVCT image performance characterization

**Figure 2:**
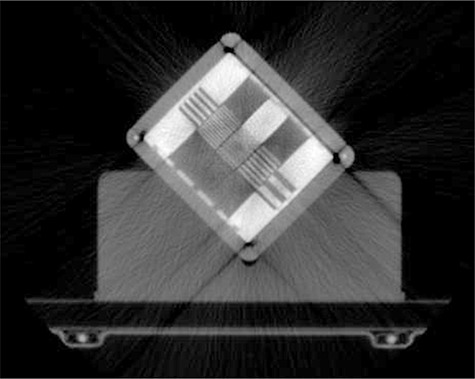
MVCT image of QC‐3 phantom.

**Figure 3:**
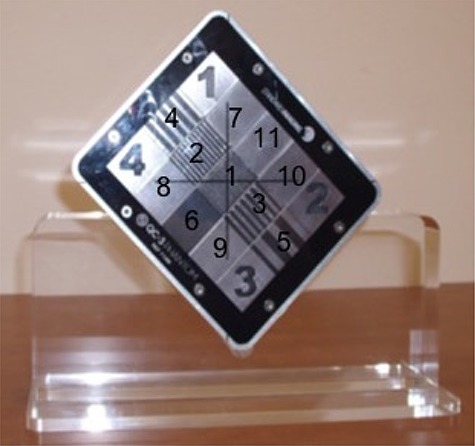
QC‐3 phantom with frame made up of aluminum and the sections made of lead and plastic.

We retrospectively studied the MVCT images (Fig. 2) acquired daily with a TomoTherapy Hi·ART II machine (TomoTherapy Inc. Madison, WI) over a period of five months. The study included a target change and a system move. MVCT image performance was characterized using PIPSpro QC‐3 phantom (Standard Imaging, Madison, WI). The QC3 phantom (Fig. 3) consists of five sets (1–5) of high‐contrast rectangular bars with spatial frequencies of 0.1, 0.2, 0.25, 0.45 and 0.76 lp/mm and bar thickness 15 mm. The frame of the phantom is made of aluminum, and the five test sections are made of lead and Delrin (Acetal) plastic (density $1.42g/{\text{cm}}^{3})$. The phantom is 15 mm thin and has 3 mm acrylic and 2 mm aluminum cover plates on the top and bottom, respectively.

The QC‐3 phantom can be used to perform the following tests: relative modulation transfer function (RMTF), spatial resolution, signal to noise ratio, and contrast to noise ratio (CNR). The use of QC‐3 phantom to determine the quality assurance of the electric portal imaging device (EPID) using the software PIPSpro, which provides the frequencies at 50%, 40% and 30% $(f_{50},\;f_{40}$ and $f_{30})$ of the maximum RMTF, has been extensively studied in literature.^(^ ^17^ ^,^ ^18^ ^)^ We have used the software to study the consistency of noise and CNR in the MVCT images acquired using TomoTherapy.

Noise, denoted as σ, is the random image noise calculated as the average standard deviation of pixel values from six different regions.

The contrast to noise ratio (CNR) as calculated by the PIPSpro software is defined as:

(3) $$\text{CNR}=(P_{b}‐P_{d})/\sigma$$

where $P_{b}$ and $P_{d}$ are the average pixel values of the brightest and darkest region of interest obtained from the uniform regions of the phantom respectively.

## III. RESULTS

### A. Stability of IVDT

**Figure 4:**
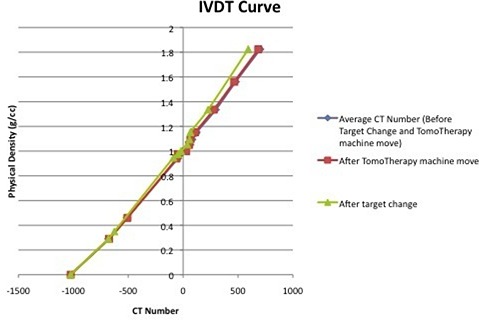
IVDT curve for average CT number taken over five months, after target changed and TomoTherapy machine moved.

The MVCT to physical density curve was established from axial scans of the “Cheese” phantom. It was noted that over a period of five months, there was no significant variation for the lower densities in the calibration curve. The normalized percent variation in HU for the low‐density plugs (less than 0.95 g/cc) was 0.72%. However, a discrepancy of 4.7% was observed in the curve at densities of $1.561g/{\text{cm}}^{3}$ and $1.824g/{\text{cm}}^{3}$ or higher. The effect of a target change and TomoTherapy machine move in the IVDT curve with respect to the IVDT curve plotted by taking an average of IVDT curve over five months is shown in Fig. 4. As there were no major upgrades in the beamline components, there was no noticeable change in the IVDT curve system move. However, a slight discrepancy for high density values is evident.

### B. Dosimetric impact of variation in IVDT

**Figure 5:**
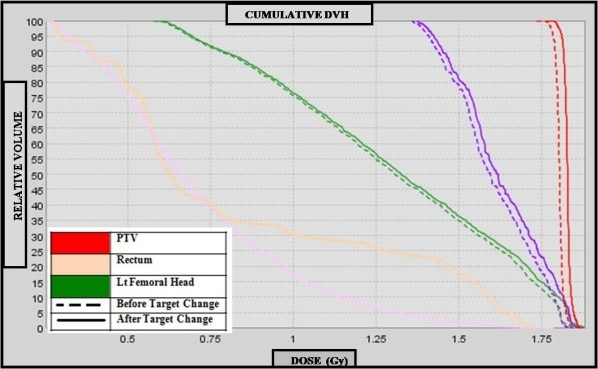
Comparison of prostate dose volume histogram before and after target change.

**Figure 6:**
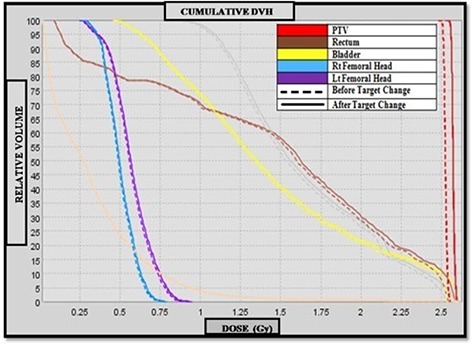
Comparison of pelvic dose volume histogram before and after target change.

The DVHs for the recalculated plans using IVDT curve before and after target change were analyzed and compared. These are illustrated in Figs. 5 and 6, respectively. A summary of the differences for the selected DVH points $\left(D_{50},\;D_{5}\right)$ for OARs is shown in Table 2 for the prostate and pelvic cases, respectively. In Table 3, pertinent dosimetric end points $(\text{HI},\;\text{TC},\;D_{99}$ and $D_{2})$ for the PTV are presented. The selected DVH points for PTVs and OARs were higher in all of the plans when calculated with IVDT curve obtained after the target change. A dose difference of 3% in $D_{99}$ and 2% in $D_{2}$ for the PTV was noted in the case of prostate. For the pelvic case, the results show that $D_{99}$ and $D_{5}$ for the PTV were 0.6% and 3% higher when curve for IVDT post‐target change was used. The largest effect of the target degradation on recalculated plans was seen for the pelvis plan where PTV is in close proximity to the femoral heads.

**Table 2:** Dose received by 50% (D50) and 5% (D5) of organ at risk, before and after target change for prostate and pelvic.

	*Organ at Risk*	*Before Target Change*		*After Target Change*		*% Difference*	
		$D_{50}(\text{Gy})$	$D_{5}(\text{Gy})$	$D_{50}(\text{Gy})$	$D_{5}(\text{Gy})$	$D_{50}$	$D_{5}$
	Rectum	43.68	70.28	43.96	71.4	0.6	1.6
Prostate	Bladder	35.84	70.84	35.98	72.8	0.4	2.7
	Rt Femoral Head	14.01	17.36	14.12	17.43	0.8	0.4
	Lt Femoral Head	14.84	21.06	14.92	21.34	0.5	1.3
Pelvic	Rectum	15.25	43.20	15.26	43.25	0.07	0.12
	Femoral Heads	31.75	44.17	31.77	45.52	0.06	3.10

**Table 3:** Target coverage (TC) and Homogeneity Index (HI) for PTV in prostate and pelvic.

	*TC and HI*	*Before Target Change*	*After Target Change*	*Difference*
	$D_{98}\%(\text{Gy})$	70.00	72.24	3.1%
	$D_{2}\%(\text{Gy})$	70.56	71.96	2.0%
Prostate‐PTV	$D_{\text{median}}(\text{Gy})$	70.28	71.12	1.2%
	HI	0.008	0.016	0.008
	TC	0.77	0.79	2.5%
Pelvic‐PTV	$D_{98}\%(\text{Gy})$	43.70	43.98	0.6%
	$D_{2}\%(\text{Gy})$	44.75	45.35	1.3%
	$D_{\text{median}}(\text{Gy})$	43.85	44.50	1.5%
	HI	0.02	0.03	0.01
	TC	0.98	0.99	1.0%

The target coverage using IVDT after target change in the case of prostate was approximately 2.5% higher than that calculated using pre‐target change IVDT. A difference of 1% was observed in target coverage for the pelvic case. The mean homogeneity index was not significantly different in either of the cases under study.

**Figure 7:**
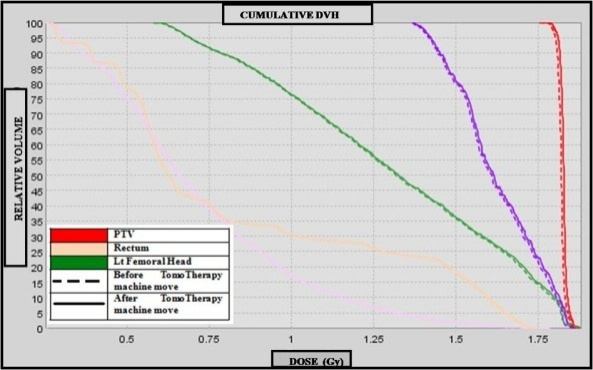
Comparison of pelvic dose volume histogram before and after TomoTherapy system move.

The effect of change in IVDT calculated before and after system move is represented in DVH as shown in Fig. 7. The DVH curves agree with each other since no significant differences were observed in the IVDT.

### C. MVCT image parameters

**Figure 8:**
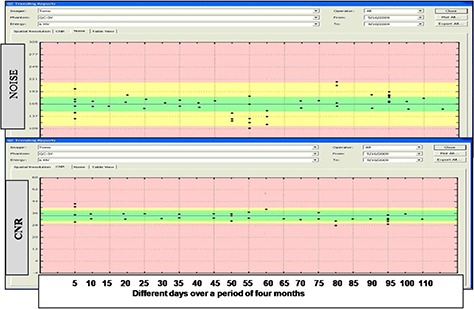
Variation of noise and contrast to noise ratio over a period of four months in the TomoTherapy machine.

The variation in noise and contrast to noise ratio are displayed in Fig. 8. The blue line is the baseline generated by taking the average of noise over a week. The baseline for CNR and noise was set to 34.5 and 64.5, respectively. The accepted deviation was set to 10% for both CNR and noise, based on the Varian linac study^(^ ^17^ ^)^ and PIPSpro manual. The green area is within acceptable parameters range; yellow area indicates caution level, and pink area indicates rejection areas, respectively. It can be noted that the noise and CNR lies in the acceptable range over the period four months.

## IV. DISCUSSION

Overall, the dose differences due to target change were all less than 5% when compared to the original plans, and exhibit the worst‐case situation just before target failure. A dose difference of less than 0.6% was observed before and after the TomoTherapy machine was moved. For treatment schedules of 30–40 fractions, a dose difference of 4%–5% for a few fractions would be considered borderline. However, if this deviation occurred during a hypo‐fractionated treatment schedule of only a few fractions, it could have the potential for a much large impact on the intended treatment. Our results indicate discrepancy, primarily in dose calculated for structures in proximity of high‐density structures. It is likely to impact specialized treatment such as the spine SBRT or total scalp treatment with whole brain avoidance. In such cases, it becomes necessary to verify the IVDT curve if MVCTs are used for adaptive planning.

## V. CONCLUSIONS

In this work, we have presented the dosimetric impact on TomoTherapy treatment plans due to variation in image value to density table (IVDT) curve as a function of target degradation. We also have investigated the reproducibility and stability of the TomoTherapy MVCT image quality over time. Multiple scans of the TomoTherapy “Cheese” phantom were performed over a period of five months. Based upon our study, we strongly recommend TomoTherapy users to record IVDT for the imaging system on a monthly basis. It is also highly important to remeasure IVDT after any major hardware repair or replacements. The IVDT curve should always be updated after target or linac change. Based on the presented results, it is clear that noise and CNR are uniformly distributed around the mean value with a variation of +5%. No significant trend in this variation was observed over the time of study. We thus recommend that measurement of noise and CNR should be incorporated into routine imaging QA on a quarterly or semi‐annual basis to check the reproducibility and stability of the TomoTherapy machine.
